# The potent neuroepithelium-promoting activity of Otx2 during gastrulation, as demonstrated by its exogenous epiblast-wide expression in chicken embryos

**DOI:** 10.3389/fcell.2025.1599287

**Published:** 2025-05-21

**Authors:** Naoya Takami, Kagayaki Kato, Koya Yoshihi, Akihito Kawamura, Hideaki Iida, Hisato Kondoh

**Affiliations:** ^1^ Faculty of Life Sciences, Kyoto Sangyo University, Kyoto, Japan; ^2^ Optics and Imaging Facility, Trans-Scale Biology Center, National Institute for Basic Biology, Okazaki, Japan; ^3^ Biohistory Research Hall, Osaka, Japan

**Keywords:** Otx2, gastrulation, neuromesodermal progenitors, chicken embryos, live imaging, RNA-seq, electroporation

## Abstract

Although Otx2 has been a topic of research for over 3 decades, the role of Otx2 expressed in the brain-forming anterior epiblast during gastrulation has not been clarified. We focused on epiblast regions where Otx2 expression is absent or downregulated, namely, the posterior epiblast and tissue belts along the bending of the neural plate. The developmental events that occurred after filling these gaps with exogenous Otx2 provided information about the roles of Otx2 during gastrulation. The following pleiotropic effects were observed: (1) development of an open and flat midbrain and hindbrain, which occurred by unzippering the previously closed neural tube; (2) precocious neural tube development at the cervical level, which resulted in thick and unclosed neural tissue; (3) absence of the sinus rhomboidalis, where neuromesodermal progenitor (NMP) stem cells multiply to provide a cell source for the trunk posterior extension; and (4) inhibition of somite development. To elucidate the principles underlying these developmental abnormalities, the cellular events were analyzed using live imaging of epiblast cells, the cell states were characterized via transcriptome analysis, and the spatial organization of transcription factor expression was determined by immunohistology. Common Otx2 activities that accounted for these pleiotropic effects were identified: (1) significant promotion of neuroepithelium development, which overrides the bipotential nature of NMPs, resulting in the loss of paraxial mesoderm precursors and multiplying NMP stem cell populations, and (2) the development of bending-refractory neural tissues. Owing to these Otx2 functions, Otx2-expressing anterior epiblast cells develop into vast amounts of brain tissue in advance of trunk neurogenesis, which occurs on a much smaller scale. We did not observe that exogenous Otx2 expression affected CNS regional specification during gastrulation.

## 1 Introduction

Otx2 is the key transcription factor for brain development, as highlighted by the lack of head tissues anterior to the ear in Otx2-knockout mouse embryos (Matsuo et al., 1995; Ang et al., 1996). During the gastrulation period of mouse and chicken embryos, Otx2 is expressed in the anterior neuroepithelium, anterior endoderm, and anterior mesendoderm (AME), among which Otx2-expressing AME was found to be the determinant of the head, including the brain, according to experiments with chimeric mouse embryos (Rhinn et al., 1998) and mouse embryos with genetically engineered AMEs (Acampora et al., 1998; Ip et al., 2014). The role of the AME as the determinant of head/brain development was also demonstrated using chicken embryos. In that study, grafting an AME underneath any anterior epiblast positions at stage (st.) 4, when gastrulation starts, elicited secondary brain development (Yoshihi et al., 2022a; Yoshihi et al., 2022b).

In mouse embryos in the stages after gastrulation (after E9.5), when basic brain architectures have been formed, Otx2 functions in the regulation of brain region-specific development (e.g., Acampora et al., 2001; Kurokawa et al., 2004), including the functional antagonism between Otx2 and Gbx2, which sets the midbrain-hindbrain boundary (Millet et al., 1996; Katahira et al., 2000; Simeone, 2000; Martinez-Barbera et al., 2001). However, these brain region-specific functions of Otx2 in postgastrulation stages cannot be extrapolated to the gastrulation period, in which Otx2 does not contribute to the specification of brain regions. At the beginning of chicken embryo gastrulation (st. 4–5), all forebrain, midbrain, and hindbrain precursors reside in the Otx2-expressing anterior epiblast (Fernandez-Garre et al., 2002; Yoshihi et al., 2022a). In addition, the subdivisioin of brain tissue, forebrain, midbrain, or hindbrain, that developed from the Otx2-expressing anterior epiblast in response to AME grafting at st. 4 depended on the graft position, indicating that gross regional specification of potential brain precursors occurred by st. 4, independent of Otx2 function (Yoshihi et al., 2022a; Yoshihi et al., 2022b).

**FIGURE 1:**
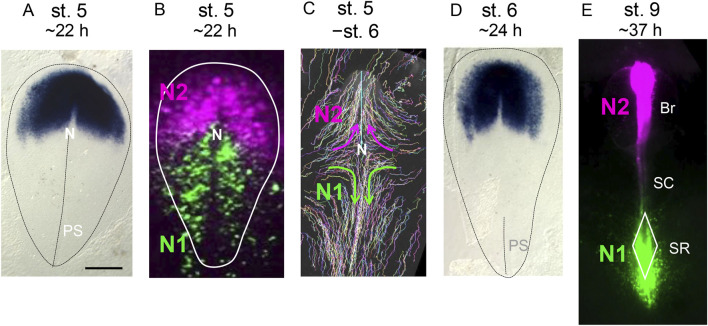
Developmental stage-dependent changes in the *Otx2* expression domains, N1 and N2 *Sox2* enhancer coverage, and cell trajectories in the gastrulation-stage epiblast. The embryonic stages were defined according to Hamburger and Hamilton (1951). Approximate periods for embryos to reach these stages under stable incubation at 38°C are indicated. The bar indicates 500 µm. **(A)** The expression profile of *Otx2* in a st. 5 chicken embryo, as determined using *in situ* hybridization. Susan Chapman produced data and deposited it in GEISHA database (Antin et al., 2014). The data was reproduced with permission from both parties. The contour of the area pellucida, which primarily develops into embryonic tissues, and the primitive streak position are indicated by the auxiliary broken lines.The anterior direction is toward the top. N, node; PS, primitive streak. **(B)** Activity of the N1 and N2 enhancers of the *Sox2* gene in stage 5 epiblast cells, as visualized by N1-EGFP and N2-mRFP vector electroporation. The N2 enhancer is active in the wide anterior epiblast area. Because N2 enhancer activation depends on the binding of Otx2 to the enhancer sequence (Iwafuchi-Doi et al., 2012), N2 activity reflects *Otx2* expression. The N1 enhancer activity initially covers the posterior epiblast broadly with minimal overlap with the N2 enhancer-active anterior epiblast. **(C)** Trajectories of epiblast cells from st. 5 to st. 6, visualized using the Supernova cell labeling vector cocktail (Yoshihi et al., 2022a). N2 enhancer-active anterior epiblast cells mostly gather at the midline and migrate anteriorly to form brain tissues. N1 enhancer-active posterior epiblast cells migrate toward the midline and then posteriorly. **(D)** The *Otx2* expression profile of a st. 6 chicken embryo, as determined using *in situ* hybridization. Susan Chapman produced data and deposited it in GEISHA database (Antin et al., 2014). *Otx2* is strongly expressed within an ∼1 mm-wide region in the anteromedial part of the epiblast, which later develops into brain tissues. Lateral to this region, a pair of *Otx2*-weak epiblast regions are formed, which later develop into the anterior surface ectoderm (Yoshihi et al., 2022a). The N2 enhancer activity at st. 6 aligns with the *Otx2* expression profile, high in the brain-forming region but low in the head ectoderm-forming region. **(E)**. Distribution of cells with N1 and N2 enhancer activity in an embryo at st. 9. Br, brain; SC, spinal cord (at this stage, the cervical spinal cord); SR, sinus rhomboidalis. The data in **(B,C)** were from Konya et al. (2024) and Nakamura et al. (2024), respectively.

What role does Otx2 play in the brain-forming anterior epiblast? Interestingly, Otx2 is not expressed in the posterior epiblast during these stages. In mouse embryos, Otx2 is expressed throughout the entire epiblast, where Otx2 in cooperation with Zic2 establishes the epiblast state primed for somatic development (Matsuda et al., 2017; Kondoh, 2024a), but as gastrulation progresses, Otx2 expression in the posterior epiblast gradually disappears (Ang et al., 1994; Martinez-Barbera et al., 2001; Iwafuchi-Doi et al., 2012). In addition, Otx2 is not expressed in the posterior epiblast in chicken embryos, presumably in response to retinoic acid signaling (Bally-Cuif et al., 1995) (Figure 1A).

In addition, when the neural plate forms and subsequently folds, the expression of *Otx2* and the related *Otx1* is downregulated along the line where the neural plate bends, initially at the midline and then at the edge (Bally-Cuif et al., 1995; Millet et al., 1996; Plouhinec et al., 2005).

We anticipated that ectopic expression of Otx2 in these typically Otx2-absent or Otx2-low epiblastic tissues would elicit abnormal developments, which would aid in deciphering Otx2 functions during the gastrulation stages. In this study, we expressed exogenous Otx2 throughout the epiblast at a physiological level to address the lack of endogenous Otx2 expression. Embryos electroporated with the vector for nonspecific Otx2 expression at the epiblast side developed pleiotropic abnormalities, which provided information about the functions of Otx2.

To understand and analyze exogenous Otx2-induced abnormalities, it is crucial to consider the regulation of Sox2, a central transcription factor involved in neural development, which differs entirely between the anterior and posterior epiblast.

Anterior to the node, *Sox2* gene transcription depends on the N2 enhancer, which is activated by the cobinding of Otx2, Zic2, and a POU factor to the enhancer sequence; therefore, transcription of this gene anterior to the node is dependent on Otx2 (Iwafuchi-Doi et al., 2012). All epiblast cells with an active N2 enhancer have the potential to develop into brain tissues; however, in normal embryogenesis, only the N2-positive cells proximal to the AME develop into brain tissues, and remote epiblasts develop into the head ectoderm (Yoshihi et al., 2022a; Yoshihi et al., 2022b; Kondoh, 2024b). In contrast, in the region posterior to the node, where Otx2 expression is absent, neural tissues largely develop from neuromesodermal progenitors (NMPs), which are characterized by N1 *Sox2* enhancer activity (Takemoto et al., 2006; Takemoto et al., 2011; Kondoh and Takemoto, 2024). These two epiblast regions with N2 enhancer activation and N1 enhancer activation do not overlap significantly (Figure 1B), which supports the model that Sox2 regulation of neural development differs entirely between the anterior and posterior epiblast.

In both the anterior and posterior epiblast fields, the cells converge to the midline from st. 5 to st. 6 (Figure 1C). N2 enhancer-positive anterior epiblast cells migrate anteriorly to develop into the brain and anterior surface ectoderm, whereas N1 enhancer-positive NMPs gathered at the midline quickly migrate posteriorly to promote posterior trunk extension (Yoshihi et al., 2022a). At st. 6, the anterior neural plate, which strongly expresses Otx2, is already formed and later develops into brain tissues, which are distinct from the low-Otx2 head surface ectoderm precursors (Figure 1D). N2 enhancer-positive cells subsequently develop into brain tissues, whereas the majority of N1 enhancer-positive NMPs accumulate in the sinus rhomboidalis tissue complex, where the stem cell population of NMPs multiply in a Wnt3a- and Bra (TbxT)-dependent manner (Yamaguchi et al., 1999; Garriock et al., 2015; Kondoh et al., 2016) and provide cell sources for trunk posterior extension (Kondoh, 2024b; Kondoh and Takemoto, 2024) (Figure 1E). At the anterior spinal cord (SC) level, the NMPs at the midline develop directly into mesodermal tissues if the cells ingress into the lower cell layer or neural tissue if the cells remain in the epiblast layer without participating in NMP stem cell production (Kondoh and Takemoto, 2024; Konya et al., 2024). Posterior to the cervical region, mesodermal tissues develop from NMP stem cells that multiply and ingress into the lower layer of the sinus rhomboidalis. Due to this NMP behavior that depends on the axial level of the cells, *Wnt3a*-knockout (Takada et al., 1994) and *Bra*-knockout (Yamaguchi et al., 1999) embryos lack tissues posterior to the forelimb while normally producing head and cervical tissues (Kondoh et al., 2016; Kondoh and Takemoto, 2024). Therefore, fully understanding the regulatory role of NMPs is essential to deciphering the pleiotropic effects of exogenous Otx2 expression on trunk development.

In this study, we identified the basic roles of Otx2 in the brain-forming anterior epiblast for the first time in 3 decades of Otx2 research. Exogenous Otx2 expression did not affect the regional specification of the CNS.

## 2 Results

### 2.1 Impact of panepiblast expression of exogenous Otx2 in chicken embryos

The mixture of the Otx2 expression vector and EGFP vector (see Materials and Methods) was electroporated during the transition from st. 4 to st. 5, abbreviated hereafter as st. 4/5 (∼19 h of incubation), when electroporation into the broad epiblast is efficient and the node has not extended AME (Yoshihi et al., 2022a). The mouse *Otx2* (*mOtx2*) cDNA sequence was used to distinguish exogenous *Otx2* from endogenous chicken *Otx2* (*cOtx2*) in the transcriptome analysis.

**FIGURE 2:**
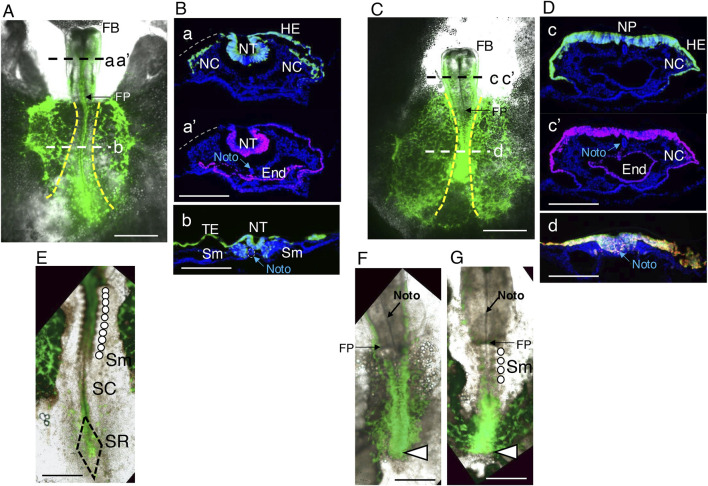
Morphological features of embryos subjected to panepiblast electroporation with exogenous Otx2 expression. Embryos with exogenous Otx2 expression were electroporated with pCAGGS-m*Otx2* and pCAGGS-EGFP vectors at st. 4/5, whereas control embryos received insert-free pCAGGS and pCAGGS-EGFP vectors; both groups of embryos were assessed for the expression of the vector-driven proteins at st. 9–10. **(A)** Control embryo. The anterior direction is toward the top. The yellow broken lines indicate the boundaries between the area pellucida (central) and the area opaca (lateral). Cryosections at axial levels a, a’ and b and immunostained for EGFP and Otx2 with nuclear DAPI staining are shown in **(B)**. FB, forebrain; FP, foregut portal. The bar indicates 1 mm. **(B)**. Immunostained cryosections of the embryo shown in **A** at the axial levels aa’ (posterior midbrain) and b (spinal cord). The bars indicate 200 μm. **a** and **a’** are the same section stained for EGFP and Otx2, with the EGFP channel fluorescence (green) and Otx2 channel fluorescence (magenta) separately overlaid on the DAPI nuclear fluorescence (blue). This section lacked the left side head ectoderm at the position indicated by the gray broken line. The epiblast-derived tissues, neural tube (NT), head ectoderm (HE), and neural crest (NC) were fully labeled with EGFP. Endogenously, Otx2 was strongly expressed in the neural tube (NT) and endoderm (E) but weakly expressed in the HE. The anterior notochord (Noto) was recognized by the DAPI staining pattern. **(b)** A section at the trunk level. The EGFP-labeled posterior epiblast cells developed into the spinal cord neural tube, trunk ectoderm (TE), and somites (Sm). No endogenous Otx2 expression was detected at this level. **(C)** Representative embryo coelectroporated for EGFP and Otx2. Although pairs of brain vesicles formed in the forebrain (FB), the midbrain and hindbrain portions developed as a continuous flat and open neural plates. In addition, the trunk portion was shortened. The bar indicates 1 mm. **(D)** Immunostained cryosections of the embryo shown in **(C)** at axial levels cc’ (midbrain) and d (trunk neural tissue). The bars indicate 200 μm. **c** and **c’** are the same section stained for EGFP and Otx2, which were individually overlaid on the DAPI nuclear fluorescence (blue). Virtually all HEs and neural plates (NPs) were simultaneously labeled with EGFP and Otx2. NC cells expressing exogenous Otx2 also developed. **(d)** A representative trunk section where the Otx2 (magenta) and EGFP (green) fluorescence signals were overlaid. The cells expressing these proteins invaginated as a thick cell belt along the trunk midline but did not migrate laterally to form the paraxial mesoderm or invaginate sufficiently to form a neural tube. The notochord was located in a ventral part of the mass of Otx2-expressing cells (see also Figure 9, below). **(E,F)** High-power images of the same embryos as in **(A,C)**, respectively, showing the details of posterior morphology and fluorescence distribution. The bars indicate 500 µm. **(E)** A normal embryo with the development of 10 pairs of somites (Sm) indicated by an array of circles, a narrow spinal cord (SC), and the sinus rhomboidalis (SN). **(F)** No somites developed, and the trunk ended abruptly (arrowhead) without forming the sinus rhomboidalis. **(G)** An embryo similar to **(F)** but with the development of four somite pairs.

The effects of exogenous Otx2 expression were first analyzed at st. 9–10 (Hamburger and Hamilton, 1951; ∼37 h of incubation) and compared with those of control embryos, which were electroporated with only the EGFP expression vector (Figures 2A,C). The electroporation of these vectors also occurred in the area opaca, extraembryonic tissues that primarily develop into the yolk sac and chorion. In the figure, the area opaca boundaries are indicated by broken yellow lines to distinguish this region from the epiblast region in the area pellucida (Figures 2A,C).

Figures 2A,B show that the neural tube, neural crest cells, surface ectoderm from the head to the trunk, and paraxial mesoderm giving rise to somites are derived from epiblast cells marked by EGFP expression. Figure 2B(a’) shows that the neural tube and anterior endoderm strongly express Otx2, whereas the head surface ectoderm moderately expresses Otx2 in normal embryos.

Figures 2C,D show the pleiotropic effects of exogenous Otx2 expression on epiblast cells. Although the forebrain (FB) developed without abnormalities, the midbrain and hindbrain portions developed as flat and open neural plates, and trunk elongation was hindered. In these embryos, EGFP was expressed in virtually all epiblast derivatives and was accompanied by Otx2 expression.

The morphological characteristics of the embryo trunks were investigated using high-magnification images (Figures 2E–G); embryos with exogenous Otx2 expression lacked the sinus rhomboidalis, the embryonic site where NMP stem cells multiply (Kondoh, 2024b; Kondoh and Takemoto, 2024). The neural tissue along the posterior embryo axis was broader than that in the ordinary spinal cord, and the posterior cell mass ended without forming the sinus rhomboidalis. In addition, somites did not form in most embryos (Figure 2F); occasionally, embryos with four somites developed. The mechanism underlying the occurrence of embryos with zero or four somite pairs is given below.

**FIGURE 3:**
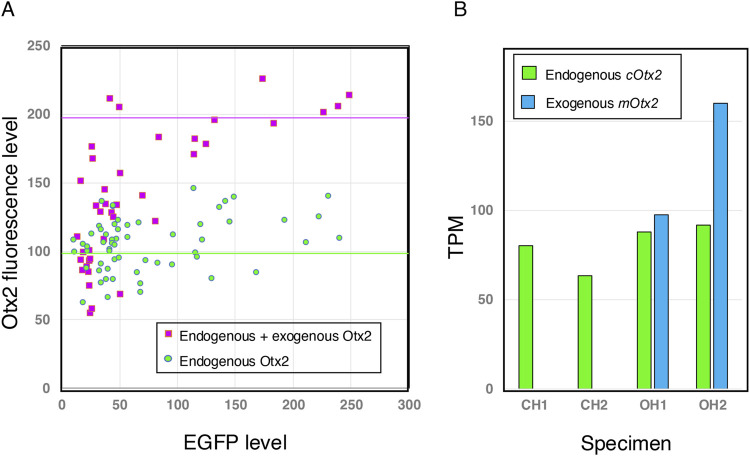
Exogenous Otx2 is expressed at a level comparable to that of endogenous Otx2 in brain tissues that develop from electroporated epiblast cells. **(A)** Brain sections immunostained for EGFP and Otx2, analogous to those in Figures 2B,D, were analyzed by measuring the fluorescence intensities of the same nuclei using Fiji (Schindelin et al., 2012). Otx2 is a highly conserved protein; mouse and chicken Otx2 differ only in one and three positions, respectively, from the immunogen human Otx2 of 289 amino acid residues. Therefore, cOtx2 and mOtx2 should be bound equally by the antibodies. Otx2 fluorescence in the low-EGFP range (<20) was assumed to be comparable in both samples (minimal contribution from exogenous Otx2) and equalized. Then, the scatter plots from both samples were compared. The exogenous Otx2 expression level, even in samples with high total Otx2 expression, was not greater than the endogenous Otx2 expression level. **(B)** Transcriptome analysis of the midbrain-hindbrain region of the brain at st.10–11, explained in Figure 7, distinguished transcripts of exogenous m*Otx2* (mouse *Otx2*) from those of endogenous c*Otx2* (chicken *Otx2*). Two embryo samples, each consisting of a pool of four embryos, were prepared for the control (chicken *Otx2* only, CH1 and CH2) and the Otx2 group (chicken and mouse *Otx2*, OH1 and OH2). TPMs averaged for four embryo samples are presented. The control embryos did not display any mouse *Otx2* transcripts. In the OH1 and OH2 samples, exogenous mouse and endogenous chicken Otx2 were expressed at comparable levels in terms of transcripts per kilobase million (TPMs).

As shown in Figure 2D, the EGFP fluorescence signal fluctuated moderately among the electroporated cells; the exogenous Otx2 level may have fluctuated similarly. Therefore, extrapolating the Otx2 level to the null EGFP level will indicate the endogenous Otx2 expression level. We measured the Otx2 nuclear fluorescence and EGFP fluorescence of the same regions of interest (ROIs) in the midbrain sections of the embryo (Figure 2B) and the corresponding regions of the flat neural plate (Figure 2D). After the Otx2 levels in these samples were equalized at the low EGFP zone, the relative Otx2 signals were plotted against the EGFP signals (Figure 3A). The average fluorescence intensity of the sum of endogenous and exogenous Otx2 was 1.37-fold greater than that of endogenous Otx2 alone (control).

When the range of EGFP fluorescence was greater than 20, indicating efficient electroporation of the cells, the Otx2 level was approximately 2-fold greater than that in the control under our experimental conditions. In the transcriptome analysis described below, the exogenous *mOtx2* transcript level was comparable to that of endogenous *cOtx2* in the midbrain-hindbrain region (Figure 3B).

Six embryos were electroporated with a 40% input of the Otx2 expression vector compared to the standard protocol. Of these, four embryos were indistinguishable from the control, while two displayed mild effects, i.e., partial opening of the midbrain and reduction in somite numbers (7 vs. 11 in controls). When Otx2 expression vector input was doubled, the electroporated embryos (5/5) displayed morphological features indistinguishable from those treated with the standard vector level reported in Figure 2. Therefore, the anterior and posterior morphological abnormalities develop with similar thresholds of exogenous Otx2 expression levels. Furthermore, these effects were saturated when the exogenous Otx2 level reached that of the standard procedure for Figure 2C.

**FIGURE 4:**
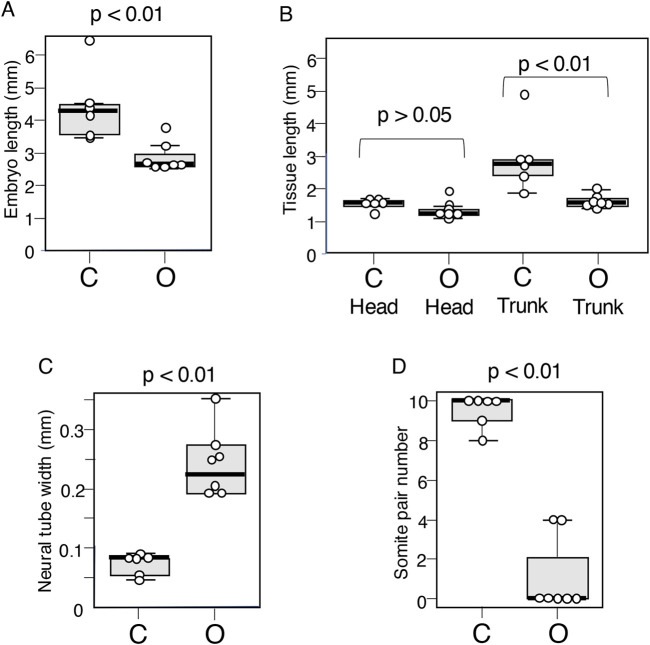
Boxplot representation of the morphological features of embryos that developed following exogenous Otx2 expression at stages 9–10. C group: Data from six control embryos. O group: Data from seven embryos with exogenous Otx2 expression. **(A)** Embryo length. Averages were 4.42 mm for C and 2.86 mm for O. *p* = 0.0066. **(B)** The lengths of the head (distance between the embryonic anterior end and the foregut portal) and the trunk (remaining embryonic portion) were assessed separately, indicating that the difference in embryo length was derived primarily from the trunk length. The average head length was 1.50 mm for C and 1.29 mm for O; *p* = 0.073. The average trunk length was 2.97 mm for C and 1.57 mm for O; *p* = 0.023. **(C)** The widths of axial neural tissues were determined using the bright-field images of the embryos. Average widths of axial neural tissue at the axial level 1 mm anterior from the embryo posterior end for the C group embryos and 0.5 mm from the posterior end for the O group embryos: 74 µm for the C group and 251 µm for the O group; *p* = 0.0026. **(D)** Somite numbers, *p* = 0.0017.

To validate the visual assessment of morphology, the following parameters were measured in six control embryos (C group) and seven embryos with exogenous Otx2 expression (O group) at st. 9–10: embryo length, head length, trunk length, trunk neural tissue width, and somite number. The results are summarized using box plots in Figure 4. The p values were determined via the Mann‒Whitney U test. The analysis confirmed that the embryo length was significantly shorter in O group embryos than in C group embryos (Figure 4A), with the primary cause being the shortened trunk (Figure 4B). The axial neural tissues (tissue architectures shown in Figures 3B,D), were significantly wider in O group embryos than in C group embryos (Figure 4C).

The O group embryos had no somite pairs (Figure 2F) or occasionally four somite pairs (Figure 2G), whereas the control embryos had ∼10 somite pairs (Figure 2E). No embryos in the experimental group presented 1–3 somite pairs (Figure 4D). This distribution of somites presumably reflected the fact that the first four somite pairs develop simultaneously (Yoshihi et al., 2022b; Kondoh, 2024b), in contrast to the sequential addition of somite pairs at later stages of development (Palmeirim et al., 1997). In other words, Otx2 expression in the posterior epiblast after st. 4 inhibits somite development, and two embryos escaped this inhibition only for the time required to develop the first four somite pairs; presumably, the subtle difference in the timing of Otx2 effectuation was responsible for embryos having either no somite pairs or four somite pairs.

In summary, the expression of exogenous Otx2 throughout the epiblast after st.4/5 resulted in pleiotropic morphological abnormalities in the embryos at st. 9–10. These abnormalities including the following: (1) Open and flat midbrain/hindbrain regions; (2) shortened trunk; (3) absence of the sinus rhomboidalis, the site of NMP stem cell multiplication, which may be correlated with the shortened trunk; (4) development of widened and unclosed neural tissue, equivalent to the spinal cord; and (5) inhibition of somite development. The following sections explore the causal processes elicited by exogenous Otx2 expression that lead to the observed morphological phenotypes.

### 2.2 Live imaging of EGFP-labeled epiblast cells to assess the dynamic impacts of exogenous Otx2 expression

**FIGURE 5:**
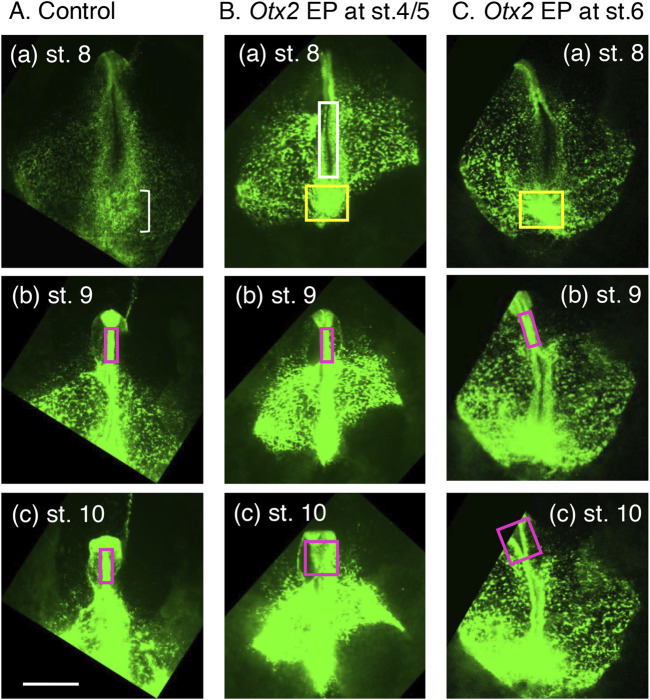
Landmark frames from Supplementary Movies S1–S3 showing the critical cellular events accounting for the morphological characteristics of embryos electroporated with the exogenous Otx2 expression vector. The image brightness was modified from the original movies. The bar indicates 1 mm. **(A)** A control embryo with electroporation of EGFP-expressing and insert-free pCAGGS vectors at st. 4/5 (∼19 h of incubation). EGFP fluorescence indicates the migration and accumulation of epiblast cells. **(a)** At st. 8, when the brain portions started to assume a neural tube, the NMP-derived spinal cord region was still a wide neural plate. The loose cell accumulation indicated by the small bracket forms the sinus rhomboidalis. **(b,c)** During st. 9 to st. 10, the midbrain and hindbrain portions remain as a narrow neural tube (magenta rectangle). **(B)** An embryo electroporated with pCAGGS-EGFP and pCAGGS-m*Otx2* at st. 4/5. **(a)** A neural tube was formed precociously at the cervical spinal cord level around st. 8 (∼29 h of incubation), as indicated by the white rectangle, and the cells accumulated at the posterior end of the trunk (yellow rectangle). **(b)** The midbrain and hindbrain portions remained as a narrow neural tube (magenta rectangle) at st. 9. **(c)** However, the midbrain and hindbrain portions were widened at st. 10. **(C)** An embryo electroporated with pCAGGS-EGFP and pCAGGS-m*Otx2* at st. 6. **(a)** The spinal cord-forming neural plate was still open, similar to the control embryo in **(A)**. However, cells accumulated at the posterior end of the trunk (yellow rectangle), similar to the embryo in **(B)**. **(b)** The midbrain and hindbrain portions remained as narrow neural tubes (magenta rectangles) at st. 9 (∼37 h of incubation). **(c)** However, the midbrain and hindbrain portions were widened (magenta rectangles) at st. 10 (∼42 h of incubation).

To investigate how exogenous Otx2 affects epiblast-derived tissue development, the collective migration of EGFP-labeled cells (i.e., cells with exogenous Otx2) in O group embryos was recorded and compared with that of the EGFP-labeled cells in control (C group) embryos (Supplementary Movies S1, S2). The epiblast cells migrated in either a medioanterior or medioposterior direction, depending on their axial level positioning (Figure 1C), regardless of exogenous Otx2 expression. However, a clear difference emerged at approximately st. 8 (∼29 h after fertilization, when the first four somites developed in control embryos). At st. 8, the posterior epiblast of control embryos exhibited a broad and uncurled neural plate (Figure 5A(a), a frame of Supplementary Movie S1); in contrast, the exogenous Otx2-expressing epiblast had already started neural tube development at the cervical level (Figure 5B(a) from Supplementary Movie S2, the region in the magenta box), suggesting a precocious occurrence of neural tissue development in the posterior epiblast with exogenous Otx2.

Supplementary Movies S1, S2 also indicated that posterior extension slowed beginning at approximately st. 8 and ceased at st. 10 in the embryos with exogenous Otx2 (Figure 5B), in contrast to the control embryos, in which posterior trunk extension continued (Figure 5A and other analogous data, e.g., a movie in Iida et al. (2020)). At this stage, a dense cell cluster (yellow box) appeared at the posterior end of the embryos expressing exogenous Otx2 (Figure 5B(a)). This cell cluster likely resulted from the premature convergence of the epiblast cells toward the midline, judged from Supplementary Movies S2, S3, where the BMP antagonists are secreted and neural development is promoted (Kondoh and Takemoto, 2024). This cell convergence developed into the thick and posterior neural tissues at st. 10 (Figure 2D(d)).

In the brain region, the midbrain and hindbrain developed as a narrow neural tube until st. 9, regardless of exogenous Otx2 expression (Figures 5A(b),B(b)). However, at st. 10, the once-closed neural tube had opened and developed into a wide flat neural plate in the embryos with exogenous Otx2 (Supplementary Movie S2; Figure 5B(c)). Therefore, wide flat neural plates, as shown in Figure 2D(c, c’), were formed only after st. 9.

### 2.3 Consequences of a later-stage supply of exogenous Otx2

The above experiments suggest that Otx2 expression in the posterior epiblast inhibits somitogenesis in a manner dependent on the timing of exogenous Otx2 expression after electroporation of the vector at st. 4/5; a fraction of the embryos that escaped this inhibition developed the first four somite pairs. According to this model, electroporation of the exogenous Otx2 vector at a later stage allows the development of a greater number of somite pairs than electroporation performed at an earlier stage but still inhibits later stages of somitogenesis.

Another critical issue is whether the posterior extension of axial tissue is suppressed even after a later stage Otx2 vector electroporation. To address this question, we electroporated the epiblast of chicken embryos at st. 6 (∼24 h of incubation), in which the brain-forming anterior epiblast region is established (Figure 1D). Six embryos electroporated at st. 6 were allowed to develop for ∼18 h, during which four control embryos reached st. 10–11 (42–45 h of incubation).

**FIGURE 6:**
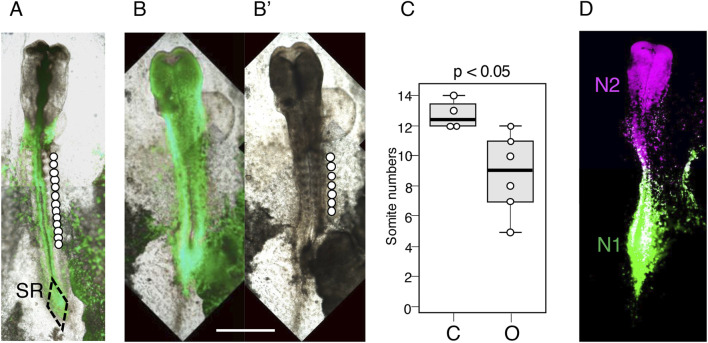
Consequences of electroporating the Otx2 expression vector at st. 6. The embryos were fixed at st. 10–11 (42–45 h of incubation). The bar indicates 1 mm. **(A)** A control embryo electroporated with pCAGGS-EGFP, with 13 somite pairs indicated by circles placed laterally to the somites. SR, sinus rhomboidalis. **(B)** An embryo electroporated with pCAGGS-EGFP and pCAGGS-m*Otx2* at st. 4, with EGFP fluorescence superimposed on the bright-field image. Note that the midbrain-hindbrain region was flattened, similar to that in embryos with electroporation at st. 4/5 (Figure 2C). **(B’)** Bright-field image of the same embryo showing the development of seven somite pairs. **(C)** Statistics of the differences in the somite numbers between four control (C) and six Otx2-electroporated (O) embryos using box plots; *p* < 0.05. **(D)** An embryo electroporated with N1-EGFP, N2-mRFP1, and CAGGS-mOtx2 vectors at st. 6 and fixed at st. 11, showing that the N1 and N2 enhancers were unaffected by exogenous Otx2 expression. N1 and N2 enhancer-active cell territories in the epiblast are maintained under exogenous Otx2 expression.

Comparing Figure 6A (control) with Figure 6B (Otx2 vector electroporation at st. 6, EGFP fluorescence superimposed on the bright-field image) and Figure 6B’ (bright-field image only) revealed the following findings: (1) The widened neural plate formed even after Otx2 vector electroporation at st. 6; (2) variable numbers of somite pairs developed (median = 9) but were significantly fewer than those of control embryos (median = 12.5, *p* = 0.017; Figure 6C); and (3) the posterior extension of thick axial neural tissue ended without the formation of sinus rhomboidalis (Figures 6B,B’), showing the same features as those of embryos electroporated at st. 4/5 (Figures 2F,G).

Live imaging of one embryo electroporated at st. 6 (Supplementary Movie S3) indicated that the posterior epiblast remained broad, similar to that of normal embryos (Figure 5C), without showing precocious neural tube development, which differs from embryos electroporated with the Otx2 vector at st. 4/5 (Figure 5B). This observation supported the model that the lack of somitogenesis in the latter embryos is due to precocious neural tube development.

Supplementary Movie S3 also revealed that a dense cell cluster formed at the posterior end of the embryos concomitantly with the midline convergence of the epiblast cells at st. 8 (Figure 5C(a), yellow box), similar to that observed after Otx2 vector electroporation at st. 4/5 (Figure 5B(a)). As discussed above regarding Figure 5B(a), this midline convergence promotes neural development. The posterior trunk extension slowed and ceased at st. 10, similar to the embryos electroporated at st. 4/5 (Figures 5B,C).

Additional embryos (*N* = 2) at st. 6 were electroporated with N2-mRFP1 and N1-EGFP vectors in addition to the Otx2 vector to examine whether exogenous Otx2 affected the activity of these enhancers. Figure 6D shows that these enhancers maintained their activity and regional coverage under exogenous Otx2 expression.

### 2.4 Transcriptome analysis of the tissues affected by exogenous Otx2 expression

**FIGURE 7:**
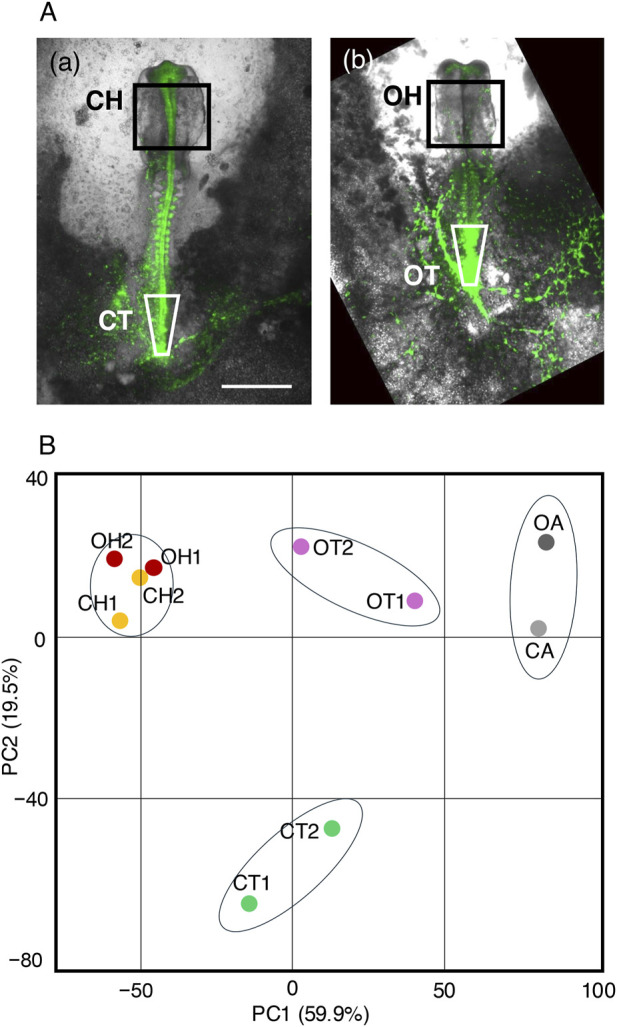
Transcriptome analysis of tissues affected by exogenous Otx2 expression via RNA-seq. **(A)** The tissues subjected to analysis in normal embryos at st. 10–11 **(a)** and in Otx2-electroporated embryos of the corresponding stages **(b)** from the same embryo pool. The bar indicates 1 mm. The CH and OH indicated by the rectangles represent the midbrain-hindbrain regions of the embryos, excluding the forebrain. The CT and OT samples in **(a,b)** are the sinus rhomboidalis in the control embryos and the corresponding region in Otx2-electroporated embryos. Tissues from four embryos were pooled to prepare a sample for RNA-seq, and duplicate samples, CH1, CH2, etc., were subjected to analysis. **(B)** PC1‒PC2 chart of the primary component analysis, showing clustering of the transcriptome profiles. Note that CH1, CH2, OH1, and OH2 constitute a tight, inseparable cluster.

To determine the mechanisms underlying exogenous Otx2-induced abnormal morphogenesis in the brain and trunk, gene transcripts in tissues from embryos electroporated with the Otx2 expression vector at st. 4/5 were compared with control embryos at st. 10–11 (∼42 h of incubation). The following tissues were collected from the control and exogenous Otx2-expressing embryos: the midbrain-hindbrain region (rectangles in Figure 7A, designated CH [a] and OH [b]) and the posterior trunk regions including the sinus rhomboidalis in the control embryos (inverted trapezoids in Figure 7A, designated CT [a] and OT [b]). The forebrain tissues unaffected by exogenous Otx2 expression were excluded from the CH/OH samples. The CT and OT samples were used to compare the sinus rhomboidalis in the control embryos and the corresponding region in the embryos with exogenous Otx2. We aimed to determine whether the gene regulatory network in the sinus rhomboidalis, which sustains the NMP stem cell expansion, still operates in the embryos with exogenous Otx2 expression that lack the sinus rhomboidalis tissue morphology. Samples from four embryos were pooled for RNA-seq analysis. Duplicate samples were prepared for these tissues and designated CH1, CH2, etc. In addition, posterior area opaca tissues from control and Otx2-electroporated embryos (CA and OA) were included to provide a tissue type distance perspective for the analysis.

Poly-A RNAs from these ten samples were subjected to RNA-seq analysis; the data were deposited in GEO with the accession number GSE292068. The data were first grouped using principal component analysis (PCA). On the PC1/PC2 chart (Figure 7B), the CT and OT samples were clustered in two remote positions along PC2, indicating a significant difference in the transcriptome profile; the OT (trunk with exogenous Otx2) group was positioned closer to the CA/OA group than the CT group was. In contrast, the CH and OH samples formed a tight, inseparable cluster, indicating that exogenous Otx2 expression did not significantly affect the gene expression profiles of the midbrain and hindbrain despite dramatic alterations in tissue morphology (Figures 2A–D).

### 2.5 Morphogenesis of the midbrain and hindbrain depends on tissue folding along the low-Otx2 axial zone

**FIGURE 8:**
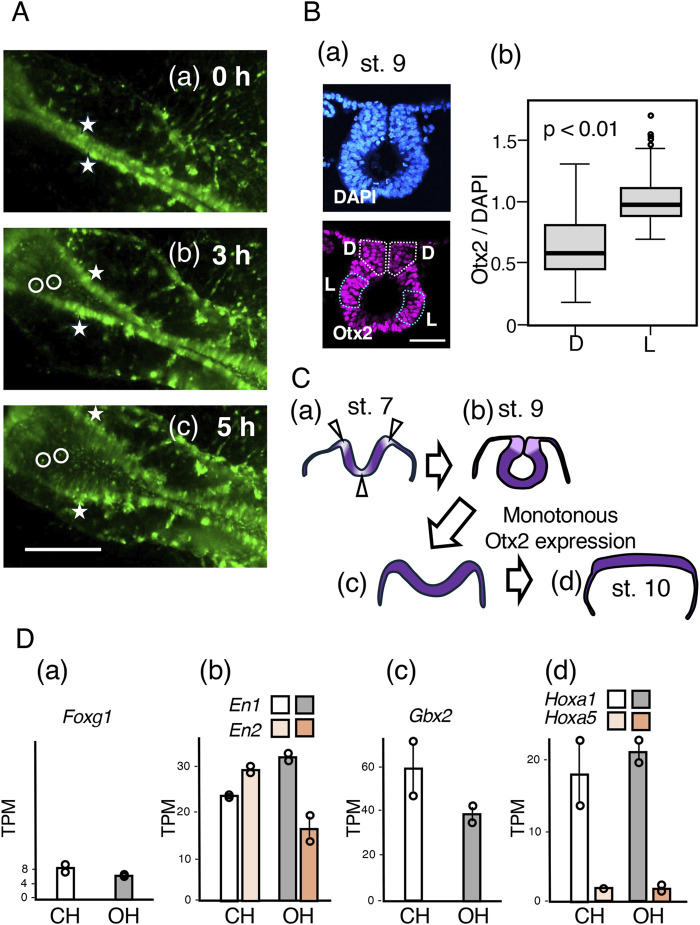
Unzippering of the closed midbrain following exogenous Otx2 expression without altering regional specification. **(A)** Time-lapse images from st. 9 to 10, covering a 5 h period, of the midbrain portion of an embryo that was electroporated with exogenous Otx2 at st. 4/5. Excerpts from the frames of Supplementary Movie S4. The bar indicates 500 µm. **(a)** St. 9 (0 h) embryo; a pair of cell clusters on the dorsal end of the closed midbrain neural tube are marked by asterisks. **(b)** After 3 h, when the opening of the dorsal ends of the neural tube had started, the cell clusters labeled by the asterisk remained on the dorsal ends. Two cells are marked on the side and at the bottom of the curled neural tube. **(c)** At 5 h, when the embryo reached st. 10, the neural tube was opened widely and flattened. The relative positions of the marked cells and cell clusters did not change **(B)**. **(a)** A cryosection of the midbrain-forming neural tube at st. 9, stained for nuclear DNA with DAPI (upper panel) and Otx2 immunofluorescence (lower panel). The dorsal area is toward the top. The bar indicates 50 μm. Otx2 expression levels were lower in the dorsal neck regions (D, outlined by white dotted lines) than in the lateral neural tube wall regions (L, outlined by blue dotted lines). **(b)** Statistics of the nuclear Otx2 immunofluorescence intensity relative to that of DAPI fluorescence, comparing the dorsal neck (D) with the lateral tube wall. A total of 120 nuclei of each dorsal neck (D) and lateral tube wall (L) in four sections at 45 µm intervals along the embryo axis were analyzed. The data indicated that the Otx2 level in the dorsal neck (D) was approximately 60% of that in the lateral tube wall (L); *p* = 3 × 10^−12^ according to Weichi’s *t*-test. **(C)** Otx2 downregulation at the bending positions of the midbrain neural plate **(a,b)** and the effect of exogenous Otx2, which opens and flattens the neural tube **(c,d)**. **(D)** The transcriptome data of the CH and OH samples collected as detailed in Figure 7 and represented as TPM. The data of duplicate samples are presented as circles, and the averages are presented as bars. The transcript levels of *Foxg1* (forebrain), *En1*/*En2* (midbrain), *Gbx2* (hindbrain), *Hoxa1* (hindbrain), and *Hoxa5* (cervical spinal cord) were not significantly affected by the exogenous expression of Otx2.

Because the gene expression profiles of the CH and OH tissues were very similar, we focused on the cellular events that occurred after exogenous Otx2 expression and led to flat and open neural plate formation. Supplementary Movie S4 and movie frames shown in Figure 8A shows that a previously closed neural tube opened in the midbrain-to-hindbrain direction within 5 h (st. 9–10). The cell clusters, two of which are marked by asterisks on the dorsal end of the neural tube (more precisely, in the “dorsal neck” in Figure 8C(a)) at st. 9 (0 h), remained on the edge of the opening sheet. Moreover, internal cells (encircled in the figure) maintained their relative positions during the 5 h period. Therefore, the opening process literally unzippered the apposed dorsal ends and flattened the previously rolled sheet.

How does exogenous Otx2 expression lead to this opening process without affecting the bulk transcriptome profile? The expression profiles of *Otx2* and functionally related *Otx1* in the midbrain region at st. 7 and st. 8 (Bally-Cuif et al., 1995; Millet et al., 1996; Plouhinec et al., 2005) indicated that the expression of these genes is reduced along the axially extended zones and the midline (ventral end) and at the junction with the surface ectoderm, where the neural plate bends. In the midbrain of st. 9 embryos, ventral bending of the neural tube had already disappeared, but the Otx2 nuclear fluorescence in the dorsal neck region closing the neural tube was significantly lower than that in the lateral neural tube wall (Figure 8B(a)). The intensity of Otx2 immunofluorescence was compared with that of 4′,6-diamidino-2-phenylindole (DAPI) fluorescence in 120 individual nuclei, confirming that Otx2 expression was reduced to approximately 60% of that in the neural tube wall (Figure 8B(b)). Thus, where Otx2 expression is reduced, the midbrain tissue is invariably bent and forms a corner. This observation indicated that an axial zone with reduced Otx2 expression provides the zone for neural plate bending along the embryo axis.

Therefore, the development of an unbent flat neural plate after exogenous Otx2 expression was likely the result of uncontrolled, monotonously high Otx2 expression abrogating the low Otx2-dependent bending of the midbrain neural tube. This inhibition occurred even after the neural tube was closed; high Otx2 expression flattened the dorsal neck of the midbrain, resulting in unzippering of the neural tube, as schematized in Figure 8C.

Despite the substantial impact of exogenous Otx2 expression on mid-hindbrain morphogenesis, the regional specificities of the neural plate were not affected. The transcriptome analysis shown in Figure 7 revealed that the expression of forebrain-specific *Foxg1* remained low (Figure 8D(a)), whereas the expression of midbrain-specific *En1*/*En2* and hindbrain-characteristic *Gbx2* under exogenous Otx2 expression (OH) was similar to that of the control (CH) midbrain-hindbrain (Figure 8D(B,C)). Moreover, the hindbrain-characteristic *Hox* genes, exemplified by *Hoxa1*, were expressed at similar levels, whereas the cervical spinal cord-specific *Hoxa5* (Nakamura et al., 2024) expression level remained very low (Figure 8D(d)).

Transcriptome analysis was performed at the st. 10–11, when the reciprocal inhibition between *Otx2* and *Gbx2* (Katahira et al., 2000; Simeone, 2000; Martinez-Barbera et al., 2001) had started to form the midbrain-hindbrain boundary eventually. The data in Figure 8D indicate that a two-fold increase, at most, in the Otx2 level in the brain-forming region (Figure 3) did not interfere with establishing brain regionality.

### 2.6 Interference in trunk posterior elongation by exogenous Otx2 expression via abrogation of sinus rhomboidalis function

**FIGURE 9:**
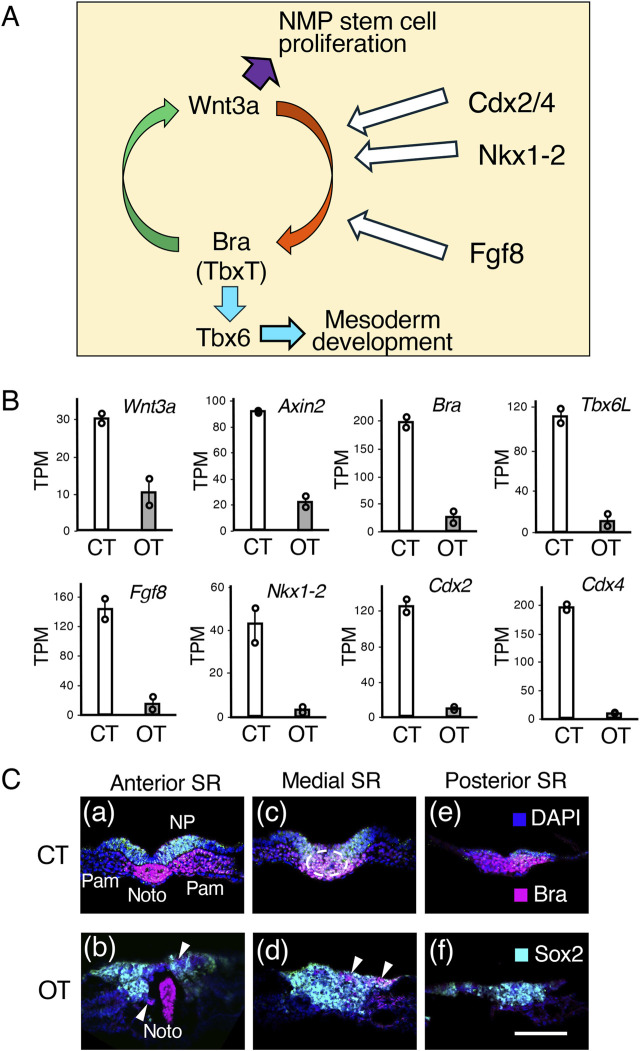
Molecular and cellular events in the sinus rhomboidalis are strongly affected by ectopic Otx2 expression in the posterior epiblast. **(A)** Wnt3a and Bra (TbxT) coregulatory loop that promotes NMP stem cell proliferation and allows (paraxial) mesodermal development via Bra→Tbx6 regulatory cascades. The activity of the transcription factors Nkx1-2, Cdx2, and Cdx4, and signaling of Fgf8, which are expressed in and around the sinus rhomboidalis, is essential for driving the Wnt3a-Bra regulatory loop and downstream processes. References are given in the main text. **(B)** Transcriptome data comparing the sinus rhomboidalis region of the control (CT) and exogenous Otx2 (OT) embryos, as indicated in Figure 7. The TPM data of duplicate samples are presented as circles, and the averages are presented as bars. The *Sox2* and *Sox1* expression levels were similar between the control embryos and those expressing exogenous Otx2 at around 19 and 1.2 TPM, respectively. **(C)** Cross cryosections of control (CT) and exogenous Otx2 (OT) embryos in the sinus rhomboidalis (SR) region immunostained for Sox2 and Bra and subjected to nuclear staining with DAPI. The panels are arranged in anterior-to-posterior order. The axial levels of the sections are indicated on top of the panels. The arrowheads in **(b,d)** indicate the small patches of Bra-expressing cells remaining in the embryos expressing exogenous Otx2. The dorsal side is toward the top. The bar indicates 100 µm. Noto, notochord; NP, neural plate; Pam, paraxial mesoderm.

In contrast to the transcript profiles of the midbrain-hindbrain region, the transcript profiles of the posterior trunk region were significantly different between controls and tissues with exogenous Otx2 expression (Figure 7B, CT1/CT2 vs OT1/OT2). Narrowing the focus of analysis to transcription factor and signaling molecule genes indicated that the expression of genes in and around the sinus rhomboidalis was significantly reduced. Figure 9A summarizes the regulatory loop sustaining NMP stem cell proliferation in the sinus rhomboidalis. The core loop comprises the mutual activation of the *Wnt3a* and *Bra* (*TbxT*) genes in the NMP population in the sinus rhomboidalis (Yamaguchi et al., 1999; Garriock et al., 2015). Downstream of this coregulatory loop, Bra activates the *Tbx6* gene [*Tbx6L* in chickens; (Knezevic et al., 1997), which promotes mesodermal development (Takemoto et al., 2011). The transcription factors Cdx2/Cdx4 (Amin et al., 2016) and Nkx1-2 (Tamashiro et al., 2012; Rodrigo Albors et al., 2018) are critically involved in driving the Wnt3a-Bra regulatory loop. Fgf8 signal input is also essential for sustaining the loop (Ciruna et al., 1997; Turner et al., 2014; Martins Costa et al., 2024).

As shown in Figure 9B, the transcript levels of *Wnt3a* and its downstream *Axin2* genes were significantly reduced (1/3–1/4) in the sinus rhomboidalis-equivalent region of Otx2-electroporated embryos. Compared with those in the control embryos, *Bra* and the coregulatory downstream mesodermal transcription factor *Tbx6L* were expressed at lower levels in Otx2-electroporated embryo trunks (<1/8). Moreover, the expression of *Fgf8* and the TF genes *Nkx2-1*, *Cdx2,* and *Cdx4* decreased even further. In brief, the sinus rhomboidalis did not develop (Figures 2E–G), and its ability to sustain the NMP stem cells was absent. The lack of the Wnt3a‒Bra coregulatory loop promoting NMP stem cell expansion was likely to be responsible for the defects in trunk posterior elongation in embryos with exogenous Otx2 expression.

The distribution of Sox2-expressing (neural-oriented) and Bra-expressing (mesodermal-oriented) cells in the sinus rhomboidalis in the control embryo and the corresponding embryonic region (judged according to the position from the trunk end and the notochord posterior end) of the embryo with exogenous Otx2 displayed marked differences (Figure 9C). At the anterior level of the sinus rhomboidalis (SR) in the control embryo (CT), the neural plate (NP, Sox2+) and bilateral paraxial mesoderm (Pam, Bra+), intervened by the notochord (Noto, Bra+), were completely separated (Figure 9C(a)). In contrast, in embryos with exogenous Otx2, the cells in the neural and mesodermal positions did not separate and expressed Sox2 (Figure 9C(b)). At the medial level of the control (CT) sinus rhomboidalis (Figure 9C(c)), the epiblast cells ingressed substantially into the mesoderm region (encircled by the broken line) and coexpressed Sox2 and Bra during the process. In the embryo with exogenous Otx2 (OT) at the same level in the region corresponding to the SR, the ingressing cells were mostly Sox2+, including a small fraction of Bra+ cells (arrowheads), (Figure 9C(b, d)). At a more posterior level, the sinus rhomboidalis in the control embryo consisted of Bra + cells (Figure 9C(d)), whereas the corresponding region in embryos with exogenous Otx2 consisted of Sox2+ neurally oriented cells (Figure 9C(e)). The same spatial distribution of Sox2 and Bra expression observed in the control embryos was reported for cells in the sinus rhomboidalis of mouse embryos (Garriock et al., 2015; Wymeersch et al., 2016).

Taken together with the transcriptome analysis shown in Figure 9B, exogenous and ectopic Otx2 expression in the posterior epiblast forced the originally bipotential NMPs at the axial position to develop into the neural pathway, inhibiting the mesoderm-producing process and the Wnt3a-Bra coregulatory loop. These inhibitions account for the loss of paraxial mesoderm (somite) development, reflecting the timing of exogenous Otx2 expression (Figures 2, 4, 6), as well as the arrest of posterior extension of the trunk by exogenous Otx2 expression (Figures 2, 4–6).

## 3 Discussion

### 3.1 Central issue of this study

Studies using genetically engineered mouse embryos have contributed critically to understanding the role of Otx2 in brain development. Otx2-knockout mouse embryos lack the head anterior to the ear (Matsuo et al., 1995; Ang et al., 1996). This dramatic phenotype was due to the lack of Otx2 expression in the anterior mesendoderm [AMS, called the visceral endoderm in early studies; (Kondoh, 2024b)] but not the Otx2-negative neuroepithelium. Examples of experimental evidence indicating the role of Otx2 in the AMS include the following: (1) In experimental chimeras of *Otx2*-positive and *Otx2*-negative cells, occupation of the AMS by *Otx2*-positive cells results in head development (Rhinn et al., 1998); (2) AME-restricted ectopic *Otx1* expression in *Otx2*-knockout embryos rescues head defects (Acampora et al., 1998); and (3) AME-specific *Otx2* ablation is sufficient for phenocopying *Otx2*-knockout embryos (Ip et al., 2014).

The involvement of Otx2 activity in the regional development of brain domains after E9.5 has been reported and discussed previously (e.g., Acampora et al., 2001; Kurokawa et al., 2004). The Otx2-Gbx2 antagonism involved in setting the midbrain-hindbrain boundary has also been highlighted (Katahira et al., 2000; Simeone, 2000; Martinez-Barbera et al., 2001). However, these regional contributions of Otx2 activity to brain development appear to follow the earlier regional specification of the neuroectoderm, a theory derived from chicken embryo studies (Yoshihi et al., 2022a; Yoshihi et al., 2022b); the earlier function of Otx2 in the developing brain during gastrulation has not been clarified.

We utilized chicken embryos in culture, which are flat, larger than mouse embryos and amenable to experiments, with greater spatial and temporal resolutions than mouse embryos (Yoshihi et al., 2022a; Yoshihi et al., 2022b). Grafting AMEs at any location underneath the Otx2-expressing epiblast at st. 4 caused the development of the second brain, supporting the conclusion about the role of the AME in brain development. At this stage, precursors of the forebrain, midbrain, and hindbrain are all included in the Otx2-expressing anterior epiblast, which is marked by N2 enhancer activity (Fernández-Garre et al., 2002; Yoshihi et al., 2022a); hence, Otx2 cannot be a player in the early-stage regional specification of the CNS. In fact, the brain tissues that develop in response to AME grafting always reflect the graft position within the Otx2-expressing epiblast territories; i.e., grafting AME at an anterior position elicits forebrain and midbrain development, whereas grafting at a posterior position results in the development of the midbrain and hindbrain (Yoshihi et al., 2022a; Yoshihi et al., 2022b), indicating that early-stage CNS regional specification occurs through a mechanism other than Otx2 action. Moreover, if Otx2 alone was a determinant of brain regionality, Otx2 would never be coexpressed with spinal cord-characteristic *Hox* genes (e.g., *Hoxa5*); however, multiple early-stage neural stem cell lines coexpressing these transcription factor genes in individual cells have been established (Nakamura et al., 2024).

What is the early-stage role of Otx2 in neuroectodermal development? We addressed this question by utilizing chicken embryos from st. 4/5 to st. 9–10, which correspond to the mouse developmental stages E7 to E9.5, the stage when Otx2 expression is reduced in the posterior region of the mouse embryo epiblast and gross brain morphology is established (Iwafuchi-Doi et al., 2012; Kondoh, 2024b). Prior to this stage, Otx2 is involved in priming epiblast cells to develop into somatic cells (Matsuda et al., 2017; Kondoh, 2024a).

### 3.2 Region-dependent morphogenetic disciplines of the neural tube, indicated by the differential impacts of exogenous Otx2 expression

#### 3.2.1 Brain morphogenesis

To understand the role of Otx2 before st. 10, we expressed exogenous Otx2 throughout the epiblast. In normal chicken embryos, posterior epiblasts lack Otx2 expression at least partly because of inhibitory retinoic acid signaling (Bally-Cuif et al., 1995). Even in anterior epiblasts expressing Otx2, the expression is diminished along the midline at st. 6 (Figure 1D) (Bally-Cuif et al., 1995; Millet et al., 1996), in which the neural plate bends concavely first, followed by bending at the dorsal ends of the midbrain and hindbrain at st. 7 to st. 8, in which the junctions to the surface ectoderm display convex bending (Millet et al., 1996; Plouhinec et al., 2005).

The underlying rationale of this experimental approach is that the consequences of ectopic or monotonous Otx2 expression in the region where Otx2 expression is diminished in normal embryos would indicate the cellular functions of Otx2 at these early developmental stages.

After exogenous Otx2 expression, the following morphogenetic abnormalities developed: (1) flat and open midbrain and hindbrain (Figures 2C,D, 6); (2) precocious anterior spinal cord development (Figure 5B); (3) decreased and arrested posterior trunk extension (Figures 2C,F,G, 4B); (4) widened axial neural tissue (Figures 2D,F,G, 4C); and (5) inhibited somitogenesis (Figures 2F,G, 4D). A critical finding when analyzing and interpreting these phenomena is that the expression level of exogenous Otx2 as a transcript or protein is comparable to that of endogenous Otx2 (Figure 3).

The development of flat open midbrain and hindbrain tissues (Figures 2C,D, 6) was a conspicuous effect of exogenous Otx2 expression. Live imaging of EGFP-labeled epiblast-derived cells (Figures 5B,C, 8A; Supplementary Movies S2–S4) revealed that midbrain/hindbrain neural tubes were originally closed but were unzippered from the midbrain to the hindbrain during the 5 h period from st. 9 to st. 10. In addition to the reduction in *Otx2* and *Otx1* expression at the neural tissue bending site in st. 7 and st. 8 (Bally-Cuif et al., 1995; Millet et al., 1996; Plouhinec et al., 2005), we found that the dorsal neck of the midbrain neural tube expressed Otx2 at a lower level (60%) than the neural tube wall did at st. 9, when the unzippering of the neural tube initiates (Figure 8B).

A likely explanation for this phenomenon is that a reduced Otx2 expression level not only elicits neural plate bending but is also essential for maintaining the bent state. The neural tube forms before exogenous Otx2 accumulates to a certain threshold; however, exogenous Otx2 expression surmounting the threshold at a later stage disrupts the maintenance of the bent state, unzipping the neural plate by the force of unbending (Figure 8C). This morphological change in the midbrain and hindbrain occurred without gross transcriptome changes (Figure 7B) while maintaining the regionalities of the brain tissues (Figure 8D). Two distinct steps in flat epithelial sheet-derived morphogenesis, initiation and maintenance, have precedence. In the *trachealess* mutant larvae of *Drosophila*, trachea development starts with the formation of epithelial pits, but the pits retract in the absence of the pit-maintaining function of *trachealess* (Kondo and Hayashi, 2019). The activity of Otx2 identified in the midbrain-hindbrain phenotype produces bending-refractory neural tissue.

Notably, forebrain development was not affected by exogenous Otx2 expression (Figures 2A,C), indicating that forebrain morphogenesis proceeds under a different mechanism from that of the midbrain and hindbrain.

It is interesting to compare the phenotype of the Otx2-deficient brain with that of the pan-epiblast Otx2 expression. A study on the *Otx2*−/−<->+/+ chimeric mouse embryos (Rhinn et al., 1998) revealed that the embryos with the wild-type ventral tissues, including AME, developed brain tissues exclusively composed of *Otx2*−/− cells. However, the neuroectoderm in the brain was thin and rounded, suggesting that tissue tension was attenuated. This *Otx2*−/− neuroectoderm phenotype is consistent with our conclusion that Otx2 promotes thick and stiff neuroectoderm development.

#### 3.2.2 Trunk posterior extension mediated by NMP stem cell proliferation

Tracing of posterior epiblast cells comprising the NMPs using DiI cell labeling indicated that cells from the anterior portion behaved distinctly from the posterior cells (Konya et al., 2024). Cells from the anterior portion migrated laterally to the midline and stayed, presumably to develop directly into the neural or mesodermal tissues. In contrast, cells in the remaining posterior portion migrated mediolaterally to the midline and then converged to the NMP stem cells of the sinus rhomboidalis, from which the neural and mesodermal tissues derived. In brief, two NMP populations exist: NMPs residing in the anterior portion and directly developing into neural and mesodermal tissues and NMPs residing in the remaining portion and developing into neural or mesodermal tissues via the NMP stem cell state.

The anteriorly positioned posterior epiblast cells with exogenous Otx2 expression closed the neural plate and developed into the neural tube precociously at st. 8 (Figure 5Ba). These epiblast cells likely represent the anterior NMP population, indicating that exogenous Otx2 strongly promoted the neural development of bipotential NMPs. Although these anteriorly positioned NMPs developed into the neural tissue, they did not form a narrow spinal cord as observed in the control embryos but remained thick and dorsally open neural tissues (Figures 2E–G), presumably because of the inclusion of too many cells in the neural tissue and the formation of bending-refractory neural tissue via the same Otx2-dependent mechanism that controls the unzippering of the midbrain-hindbrain neural tube. The inhibition of initial somitogenesis in embryos with exogenous Otx2 expression is explained by the exhaustion of the NMP pool, which would otherwise have contributed to somitogenesis.

The NMP derivatives at the posterior end of the embryo trunk were strongly affected by the morphology, namely, the absence of the sinus rhomboidalis structure (Figures 2E–G), and the transcriptome profile (Figure 4). Transcriptome analysis revealed that all genes involved in the sustained multiplication of NMP stem cells (Figure 9A), which occurs in the sinus rhomboidalis, were downregulated (Figure 9B); this observation correlated with the transcriptome abnormalities and morphological changes.

Analysis of the distribution of Sox2-expressing cells (proneural) and Bra-expressing cells (promesodermal) by immunostaining of sinus rhomboidalis sections from control embryos revealed that Sox2-expressing neural plates and bilateral Bra-expressing paraxial mesoderm intervened by the notochord are completely segregated at the anterior level (Figure 9C(a)), cells coexpressing Sox2 and Bra predominate among epiblast cells that ingress into the mesodermal layer (Figure 9C(c)), and cells at the posterior end mostly express Bra (Figure 9C(e)), as expected for the sinus rhomboidalis tissue (Kondoh, 2024b; Kondoh and Takemoto, 2024).

In contrast, most epiblast-derived tissues in embryos with exogenous Otx2 expressed only Sox2 at all corresponding axial levels, and the Bra-expressing minority cells did not segregate from the Sox2--expressing cell mass (Figure 9C). The Sox2-expressing cells appeared to have ingressed into the mesodermal layer but maintained Sox2 expression without Bra activation; these cells were not segregated from the cells of the upper surface and did not migrate laterally as is typical of paraxial mesoderm-producing cells (Figure 9C(b,d)). In addition, the cells at the posterior end expressed Sox2 (Figure 9C(f)), in contrast to the Bra dominance at the corresponding axial level of the control embryos (Figure 9C(e)).

Overall, the above observations indicate that neither the sinus rhomboidalis tissues nor proliferating NMP stem cells are present in the posterior epiblast of the embryos expressing ectopic Otx2. Based on this observation, two questions arise: (1) Does exogenous Otx2 directly affect the proliferation of NMP stem cells? Or (2) does it force all resident NMPs to undergo neural development, exhausting the NMP population? We feel the second model is more likely because if Otx2 directly targets the self-renewal of NMP stem cells, more than four pairs of somites would have been produced before the posterior trunk extension was arrested. Furthermore, the latter model is also supported by the observation that Otx2-expressing posterior epiblast cells accumulate toward the midline, the site where the neural development is promoted, before the trunk posterior extension halts (Figures 5B(a),C(a)). However, the first model cannot be excluded formally.

### 3.3 Role of Otx2 in neural development during the gastrulation period

We investigated the effects of panepiblast Otx2 expression on the development of epiblast-derived tissues, mainly neural tissues, during the gastrulation period (st. 5–10) in chicken embryos. The effects appeared to be pleiotropic, with a dependence on the axial levels. We investigated the individual phenomena by combining live imaging, transcriptome analysis, and immunostaining of embryonic tissues.

The final outputs of exogenous Otx2 varied depending on the axial level, reflecting the region-dependent morphogenetic processes of the neural tube. However, common denominators across the phenomena were identified: (1) the promotion of neurogenesis, which overrides even the bipotentiality of NMPs, without specifying the CNS domains and (2) the development of bending-refractory neural tissues.

When gastrulation initiates, Otx2 expression is confined to the anterior epiblast in both chicken and mouse embryos (Figure 1A). This localization of Otx2 guarantees that the brain develops first, preceding the sequential posterior addition of trunk spinal cord tissues. Owing to the neurogenesis-promoting activity of Otx2, ectopic Otx2 expression promoted prcocious neural development at the cervical level (Figure 5B(a)).

Conversely, the absence of Otx2 activity in the posterior epiblast is essential for the development of trunk tissue, which depends on bipotential NMPs as the tissue source. Otherwise, as observed in embryos with exogenous Otx2 expression, the majority of NMPs develop into neural tissues expressing Sox2, exhausting the Bra-expressing cells required for paraxial mesoderm development and NMP stem cell multiplication (Figure 9).

Unlike the NMP-derived tissues, the notochord developed normally in the embryos expressing exogenous Otx2 (Figures 2F,G). The expression of Brachyury in the notochord of these embryos was comparable to that of the control embryos (Figure 9C). Contrastingly, most of the NMP-derived cells expressed Sox2, leaving only small patches of cells expressing Brachyury (Figures 9C(b,c), arrowheads).

The second effect of Otx2 that leads to the development of a stiff, flat neural tube, if unregulated, provides new insight into how brain morphogenesis is regulated. The Otx2 expression level must be finely tuned locally, at least at the midbrain-hindbrain level. Our study also emphasized the value of live imaging analysis of embryonic development in addressing similar issues (Supplementary Movies S1–S4).

As shown in Figure 1, neurogenic Sox2 is differentially regulated in the anterior and posterior epiblasts. Sox2 is activated in a broad area in the anterior epiblast owing to the Otx2-dependent N2 enhancer (Iwafuchi-Doi et al., 2012). As discussed above, the large brain tissues develop quickly due to the massive accumulation of N2 enhancer-high epiblasts (Yoshihi et al., 2022b), combined with the neurogenic Otx2 activity. In the posterior epiblast, the N1 enhancer marking the NMPs regulates the initial step of neurogenesis (Takemoto et al., 2006; Kondoh and Takemoto, 2024). In the regions distant from the midline, the N1 enhancer is activated but its action is idle partly due to the inhibition of *Sox2* expression by BMP signaling before convergence to the midline, where BMP inhibitors are secreted. Following the convergence to the midline, the NMPs develop into the neural or mesodermal tissues directly at the cervical level, as discussed in Figure 5B(a), or develop into NMP stem cells at the thoracic level that sustain the posterior trunk extension (Kondoh and Takemoto, 2024). As demonstrated in this study, Otx2 expression in the posterior epiblast interferes with proper NMP development, possibly because Otx2 activity preferentially promotes neural development. In short, the lack of Otx2 in the posterior epiblast enables proper spatial switch of Sox2 enhancer usage, N2 in the anterior epiblast to N1 in the posterior epiblast, respectively, guaranteeing expanded brain development and proper body extension along with neural and mesodermal development.

## 4 Materials and methods

### 4.1 Chicken embryo culture and electroporation

Fertile chicken eggs were obtained from a local supplier (Yamagishi, Japan) and incubated at 38°C to the developmental stage at the transition from stage (st.) 4 to st. 5, abbreviated as st. 4/5 in this study, or to st. 6, according to Hamburger and Hamilton (1951). The Otx2 expression vector pCAGGS-m*Otx2*, carrying the mouse *Otx2* cDNA sequence (Iwafuchi-Doi et al., 2012), at 0.25 µg and the control vector pCAGGS-EGFP at 0.2 µg in 1 µL of 10 mM Tris/HCl and 1 mM ethylenediaminetetraacetic acid at pH 7.5 were injected into the space between the epiblast and the vitelline membrane and electroporated using the standard procedure (Uchikawa et al., 2003; Uchikawa et al., 2017). For control embryos, pCAGGS-mOtx2 was replaced with the insert-free CAGGS vector. The embryos were subsequently cultured on the ventral side in an upward orientation (Konya et al., 2024) for 18–22 h until they reached st. 9–11.

### 4.2 Live imaging of embryos with EGFP-labeled epiblast cells

The embryos electroporated with the vectors described above were subjected to live imaging at intervals of 10 min using an EVOS M5000 inverted microscope (Thermo Fisher) equipped with an Olympus/Evident ×2 PLAPON objective and an onstage incubator. Because the embryo images drifted on the X–Y plane during imaging, they were aligned to a constant embryo axis with a fixed head position. To define the embryo axis in each frame, we developed a custom ImageJ plugin to manually annotate the head and trunk fixed positions. The resulting head–trunk vectors were then used to translate and rotate each frame using in-house R scripts and C applications, aligning the head to a fixed position and consistently orienting the anterior–posterior axis (Yoshihi et al., 2022a).

### 4.3 Tissue cryosection immunostaining

Embryos were fixed with 4% paraformaldehyde in phosphate-buffered saline (Nacalai Tesque) at 4°C overnight and preserved in methanol at −20°C. The samples were rehydrated and embedded in 30% sucrose/OCT compound (Sakura) (1:2), and frozen sections with a thickness of 15 µm were prepared using a Bright OTF cryostat. The cryosections were stained with the following primary antibodies: anti-GFP rabbit IgG (MBL 598, 1/1000), anti-human Otx2 goat IgG (R&D AF1979, 1/100), anti-human Bra goat IgG (R&D AF2085, 1/100), and anti-mouse Sox2 rabbit IgG (MBL PM056, 1/500). The following secondary antibodies were used in combination: anti-rabbit IgG donkey IgG-Alexa488 (abcam ab150077, 1/200), anti-goat IgG donkey IgG-Alexa568 (abcam ab175704, 1/200) and anti-rabbit IgG donkey IgG-Alexa647 (abcam ab150075, 1/200). Nuclei in the sections were stained with DAPI (1 μg/mL). Images were captured using an EVOS M5000, Axioplan 2 (Zeiss), and TCS-SPE laser microscope (Leica).

### 4.4 Transcriptome analysis

Total RNA was isolated from the tissues of four pooled embryos at st. 10–11 using TRIzol reagent (Thermo Fisher). The tissues subjected to analysis were the midbrain-hindbrain region (in duplicate), the sinus rhomboidalis and corresponding embryonic region (in duplicate), and the posterior area opaca of the control embryos and embryos with exogenous Otx2 expression. Libraries for mRNA sequence reads were prepared using a TruSeq Etrant mRNA LT Sample Prep Kit, and 100 bp paired-end reads were obtained with NovaSeq X. The transcript reads were mapped to the chicken genome using NCBI_Assembly. GCF_016700215.2 bGalGal1.pat.whiteleghornlayer.GRCg7w was used as the reference, with annotation by NCBI *Gallus gallus* Annotation Release 106. The gene transcripts were assembled with StringTie, and the gene expression profile was reported as transcripts per kilobase million (TPM) values for each sample. The original and processed data were deposited to GEO with the accession number GSE292068.

### 4.5 Optical data processing and statistical data analysis

Microscopy images were processed using Fiji (Schindelin et al., 2012), and statistical data analyses were performed via Excel (Microsoft) and R software (R Core Team, 2021).

## Data Availability

The datasets presented in this study can be found in online repositories. The names of the repository/repositories and accession number(s) can be found below: NCBI GEO under GSE292068.
