# Supplementary material for: The potent neuroepithelium-promoting activity of Otx2 during gastrulation, as demonstrated by its exogenous epiblast-wide expression in chicken embryos
Source: Front Cell Dev Biol. 2025 May 21;13:1599287. doi: 10.3389/fcell.2025.1599287 (PMC12133752; doi:10.3389/fcell.2025.1599287)
Supplement: Supplementary file 4 [file DataSheet1.docx]

Movie legends

**Movies 1** to **3** present time-lapse recordings of the migration of EGFP-labeled epiblast cells after electroporation with pCAGGS-EGFP. The fluorescence images were captured at 10-minute intervals, and the play speed was set at 6 frames per second. Therefore, one second in the movie represents one hour in real time. The CAGGS enhancer-driven fluorescence increased with time; the fluorescence brightness in the movie was set for optimum visualization of cell migration from st. 6 (~24 h of incubation) to st. 8 (~30 h); in the later stage, movie frames lost resolution owing to halation. Please see **Figure 5A** to **C** for better photo panels for the stages after st. 8 and for each movie's feature points. The movie frame width corresponds to 5 mm.

**Movie 1**. Record of a control embryo electroporated with pCAGGS-EGFP and an insert-free pCAGGS vector at st. 4/5 (~19 h of incubation), representing the migration of normal epiblast cells after st. 6, when fluorescence became detectable.

**Movie 2**. An embryo electroporated with pCAGGS-EGFP (for cell labeling) and pCAGGS-mOtx2 at st. 4/5 (~19 h of incubation) showing the following features: precocious neural tube formation at the spinal cord level (approximately st. 8, ~29 h of incubation), slowing of posterior extension of the trunk (after st. 8), and widening of the midbrain and hindbrain portions (approximately st. 9, ~37 h of incubation).

**Movie 3**: An embryo electroporated with pCAGGS-EGFP (for cell labeling) and pCAGGS-mOtx2 at st. 6 (~24 h of incubation). The spinal cord-forming neural plate was still wide open, similar to that of the control embryo (**Movie 1**); however, posterior trunk extension slowed after st. 8, similar to the embryos electroporated at st. 4/5 (**Movie 2**), and the midbrain portion widened at st. 10.

**Movie 4**. Fluorescence recording of the midbrain-hindbrain region of an embryo electroporated with pCAGGS-EGFP (for cell labeling) and pCAGGS-mOtx2 at st. 4/5 from st. 9 (~37 h of incubation) to st. 10 (~42 h of incubation). Fluorescence images captured at 10-minute intervals are shown at 3 frames per second. Unzippering of the once closed midbrain-hindbrain region is shown. The movie frame width corresponds to 1.37 mm.
